# Supplementary material for: Non-menstrual pelvic symptoms and women’s quality of life: a cross-sectional observational study
Source: PLoS One. 2025 Apr 29;20(4):e0321922. doi: 10.1371/journal.pone.0321922 (PMC12040126; doi:10.1371/journal.pone.0321922)
Supplement: S2 File — (PDF) [file pone.0321922.s002.pdf]

Explore

Case Processing Summary

	Cases					
	Valid		Missing		Total	
	N	Percent	N	Percent	N	Percent
SF36PF	369	100.0%	0	0.0%	369	100.0%
SF36PRF	369	100.0%	0	0.0%	369	100.0%
SF36BP	369	100.0%	0	0.0%	369	100.0%
SF36GHP	369	100.0%	0	0.0%	369	100.0%
SF36VIT	369	100.0%	0	0.0%	369	100.0%
SF36SRF	369	100.0%	0	0.0%	369	100.0%
SF36ERF	369	100.0%	0	0.0%	369	100.0%
SF36MH	369	100.0%	0	0.0%	369	100.0%
EHP30Pain	282	76.4%	87	23.6%	369	100.0%
EHP30CPower	282	76.4%	87	23.6%	369	100.0%
EHP30SSupp	282	76.4%	87	23.6%	369	100.0%
EHP30EWbeing	282	76.4%	87	23.6%	369	100.0%
EHP30SImage	282	76.4%	87	23.6%	369	100.0%
EHP30Sex	261	70.7%	108	29.3%	369	100.0%
EHP30Work	216	58.5%	153	41.5%	369	100.0%
EHP30FMed	264	71.5%	105	28.5%	369	100.0%
EHP30Infert	223	60.4%	146	39.6%	369	100.0%
EHP30RChild	73	19.8%	296	80.2%	369	100.0%
EHP30FTreat	208	56.4%	161	43.6%	369	100.0%
DDysp	357	96.7%	12	3.3%	369	100.0%
NMPPain	369	100.0%	0	0.0%	369	100.0%
NMDysch	368	99.7%	1	0.3%	369	100.0%
NMDysur	367	99.5%	2	0.5%	369	100.0%

Tests of Normality

	Kolmogorov-Smirnov[a]			Shapiro-Wilk		
	Statistic	df	Sig.	Statistic	df	Sig.
SF36PF	.157	369	<.001	.886	369	<.001
SF36PRF	.250	369	<.001	.772	369	<.001
SF36BP	.133	369	<.001	.955	369	<.001
SF36GHP	.103	369	<.001	.968	369	<.001
SF36VIT	.079	369	<.001	.971	369	<.001
SF36SRF	.124	369	<.001	.945	369	<.001
SF36ERF	.274	369	<.001	.757	369	<.001
SF36MH	.068	369	<.001	.982	369	<.001
EHP30Pain	.115	282	<.001	.942	282	<.001
EHP30CPower	.097	282	<.001	.936	282	<.001
EHP30SSupp	.098	282	<.001	.947	282	<.001
EHP30EWbeing	.060	282	.017	.980	282	<.001
EHP30SImage	.122	282	<.001	.929	282	<.001
EHP30Sex	.092	261	<.001	.932	261	<.001
EHP30Work	.156	216	<.001	.893	216	<.001
EHP30FMed	.296	264	<.001	.756	264	<.001
EHP30Infert	.096	223	<.001	.947	223	<.001
EHP30RChild	.180	73	<.001	.893	73	<.001
EHP30FTreat	.109	208	<.001	.948	208	<.001
DDysp	.278	357	<.001	.822	357	<.001
NMPPain	.220	369	<.001	.869	369	<.001
NMDysch	.454	368	<.001	.587	368	<.001
NMDysur	.517	367	<.001	.353	367	<.001

a. Lilliefors Significance Correction

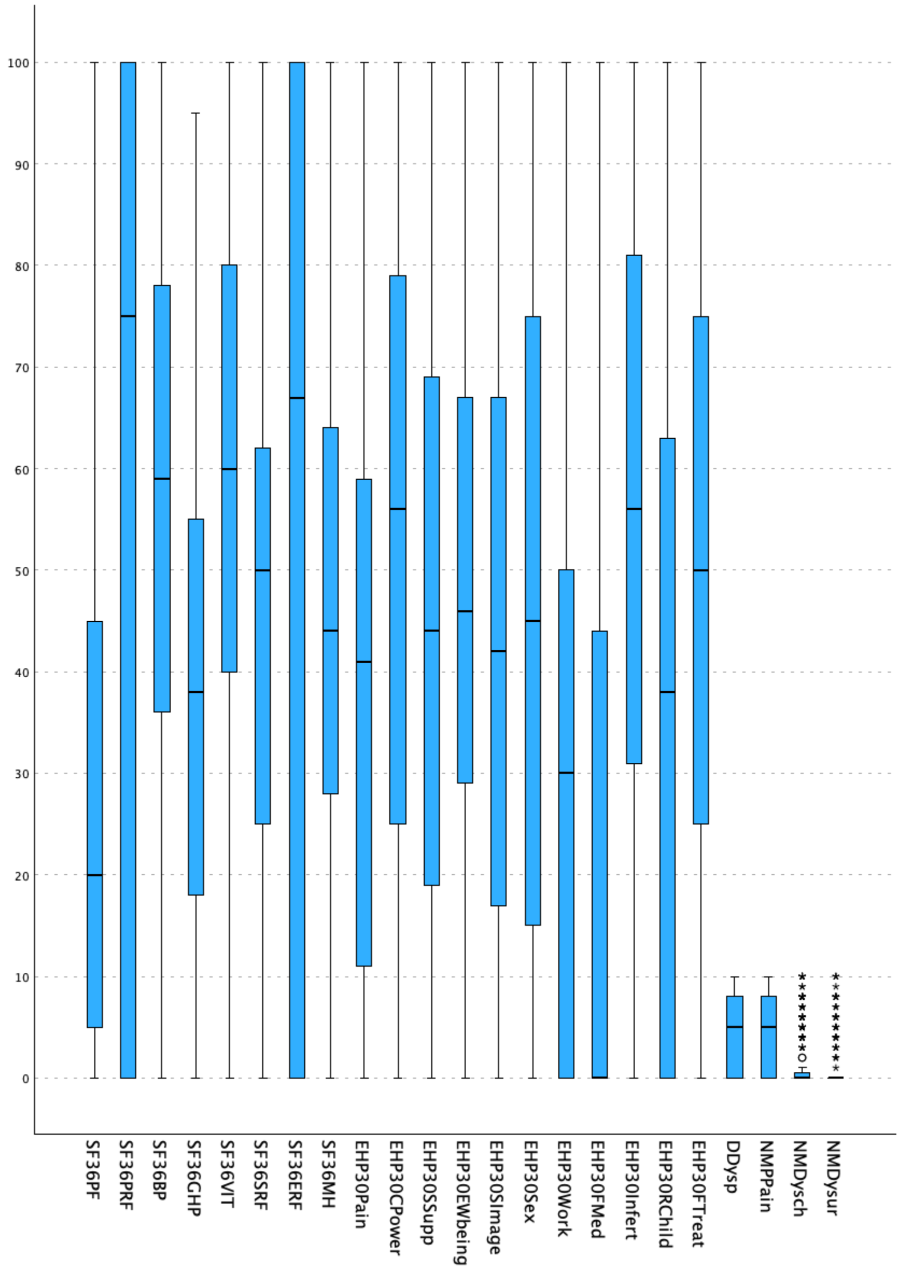

100
90
80
70
60
50
40
30
20
10
0
SF36PF
SF36PRF
SF36BP
SF36GHP
SF36VIT
SF36SRF
SF36ERF
SF36MH
EHP30Pain
EHP30CPower
EHP30SSupp
EHP30EWbeing
EHP30SImage
EHP30Sex
EHP30Work
EHP30FMed
EHP30Infert
EHP30RChild
EHP30FTreat
DDysp
NMPPain
NMDysch
NMDysur

Regression SF36PF

Variables Entered/Removed[a]

Model	Variables Entered	Variables Removed	Method
1	NMDysur, DDysp, NMPPain, NMDysch[b]	.	Enter

a. Dependent Variable: SF36PF
b. All requested variables entered.

Model Summary

Model	R	R Square	Adjusted R Square	Std. Error of the Estimate
1	.443[a]	.197	.187	22.648

a. Predictors: (Constant), NMDysur, DDysp, NMPPain, NMDysch

ANOVA[a]

Model		Sum of Squares	df	Mean Square	F	Sig.
1	Regression	43826.297	4	10956.574	21.361	<.001[b]
	Residual	179006.542	349	512.913		
	Total	222832.839	353			

a. Dependent Variable: SF36PF
b. Predictors: (Constant), NMDysur, DDysp, NMPPain, NMDysch

Coefficients[a]

		Unstandardized Coefficients		Standardized Coefficients			Collinearity Statistics	
Model		B	Std. Error	Beta	t	Sig.	Tolerance	VIF
1	(Constant)	12.856	2.079		6.185	<.001		
	DDysp	.912	.336	.139	2.713	.007	.874	1.144
	NMPPain	1.509	.371	.213	4.071	<.001	.842	1.187
	NMDysch	2.199	.475	.243	4.626	<.001	.835	1.197
	NMDysur	.297	.670	.022	.443	.658	.917	1.091

a. Dependent Variable: SF36PF

Bootstrap for Coefficients

Model		B	Bootstrap[a] Bias	Std. Error	Sig. (2-tailed)	95% Confidence Interval Lower	Upper
1	(Constant)	12.856	.028	1.878	<.001	9.178	16.686
	DDysp	.912	-.011	.359	.009	.253	1.664
	NMPPain	1.509	.016	.382	<.001	.766	2.343
	NMDysch	2.199	-.030	.532	<.001	1.105	3.232
	NMDysur	.297	-.022	.725	.670	-1.130	1.693

a. Unless otherwise noted, bootstrap results are based on 1000 bootstrap samples

Collinearity Diagnostics[a]

Model	Dimension	Eigenvalue	Condition Index	Variance Proportions (Constant)	DDysp	NMPPain	NMDysch	NMDysur
1	1	3.066	1.000	.03	.03	.03	.04	.02
	2	.839	1.911	.03	.03	.02	.05	.76
	3	.564	2.331	.03	.02	.00	.88	.22
	4	.312	3.135	.07	.85	.31	.00	.00
	5	.219	3.741	.83	.07	.64	.04	.00

a. Dependent Variable: SF36PF

Regression SF36PRF

Variables Entered/Removed[a]

Model	Variables Entered	Variables Removed	Method
1	NMDysur, DDysp, NMPPain, NMDysch[b]	.	Enter

a. Dependent Variable: SF36PRF
b. All requested variables entered.

Model Summary

Model	R	R Square	Adjusted R Square	Std. Error of the Estimate
1	.428[a]	.183	.174	39.492

a. Predictors: (Constant), NMDysur, DDysp, NMPPain, NMDysch

ANOVA[a]

Model		Sum of Squares	df	Mean Square	F	Sig.
1	Regression	122101.403	4	30525.351	19.572	<.001[b]
	Residual	544305.729	349	1559.615		
	Total	666407.133	353			

a. Dependent Variable: SF36PRF
b. Predictors: (Constant), NMDysur, DDysp, NMPPain, NMDysch

Coefficients[a]

		Unstandardized Coefficients		Standardized Coefficients			Collinearity Statistics	
Model		B	Std. Error	Beta	t	Sig.	Tolerance	VIF
1	(Constant)	32.816	3.625		9.054	<.001		
	DDysp	.994	.586	.088	1.696	.091	.874	1.144
	NMPPain	3.034	.646	.247	4.694	<.001	.842	1.187
	NMDysch	2.967	.829	.189	3.579	<.001	.835	1.197
	NMDysur	2.133	1.169	.092	1.825	.069	.917	1.091

a. Dependent Variable: SF36PRF

Bootstrap for Coefficients

Model		B	Bootstrap[a] Bias	Std. Error	Sig. (2-tailed)	95% Confidence Interval Lower	Upper
1	(Constant)	32.816	.142	3.679	<.001	25.414	40.112
	DDysp	.994	.017	.596	.093	-.137	2.217
	NMPPain	3.034	-.041	.701	<.001	1.596	4.407
	NMDysch	2.967	.016	.701	<.001	1.474	4.322
	NMDysur	2.133	.007	.935	.022	.369	4.002

a. Unless otherwise noted, bootstrap results are based on 1000 bootstrap samples

Collinearity Diagnostics[a]

Model	Dimension	Eigenvalue	Condition Index	Variance Proportions (Constant)	DDysp	NMPPain	NMDysch	NMDysur
1	1	3.066	1.000	.03	.03	.03	.04	.02
	2	.839	1.911	.03	.03	.02	.05	.76
	3	.564	2.331	.03	.02	.00	.88	.22
	4	.312	3.135	.07	.85	.31	.00	.00
	5	.219	3.741	.83	.07	.64	.04	.00

a. Dependent Variable: SF36PRF

Regression SF36BP

Variables Entered/Removed[a]

Model	Variables Entered	Variables Removed	Method
1	NMDysur, DDysp, NMPPain, NMDysch[b]	.	Enter

a. Dependent Variable: SF36BP
b. All requested variables entered.

Model Summary

Model	R	R Square	Adjusted R Square	Std. Error of the Estimate
1	.514[a]	.264	.256	22.788

a. Predictors: (Constant), NMDysur, DDysp, NMPPain, NMDysch

ANOVA[a]

Model		Sum of Squares	df	Mean Square	F	Sig.
1	Regression	65140.437	4	16285.109	31.362	<.001[b]
	Residual	181225.314	349	519.270		
	Total	246365.751	353			

a. Dependent Variable: SF36BP
b. Predictors: (Constant), NMDysur, DDysp, NMPPain, NMDysch

Coefficients[a]

		Unstandardized Coefficients		Standardized Coefficients			Collinearity Statistics	
Model		B	Std. Error	Beta	t	Sig.	Tolerance	VIF
1	(Constant)	34.092	2.091		16.301	<.001		
	DDysp	1.101	.338	.160	3.255	.001	.874	1.144
	NMPPain	2.270	.373	.305	6.089	<.001	.842	1.187
	NMDysch	1.995	.478	.210	4.172	<.001	.835	1.197
	NMDysur	.717	.674	.051	1.063	.289	.917	1.091

a. Dependent Variable: SF36BP

Bootstrap for Coefficients

Model		B	Bootstrap[a] Bias	Std. Error	Sig. (2-tailed)	95% Confidence Interval Lower	Upper
1	(Constant)	34.092	.061	2.198	<.001	30.022	38.728
	DDysp	1.101	-.004	.356	.003	.374	1.745
	NMPPain	2.270	.007	.394	<.001	1.472	3.065
	NMDysch	1.995	-.019	.429	<.001	1.141	2.821
	NMDysur	.717	.004	.548	.186	-.299	1.830

a. Unless otherwise noted, bootstrap results are based on 1000 bootstrap samples

Collinearity Diagnostics[a]

Model	Dimension	Eigenvalue	Condition Index	Variance Proportions (Constant)	DDysp	NMPPain	NMDysch	NMDysur
1	1	3.066	1.000	.03	.03	.03	.04	.02
	2	.839	1.911	.03	.03	.02	.05	.76
	3	.564	2.331	.03	.02	.00	.88	.22
	4	.312	3.135	.07	.85	.31	.00	.00
	5	.219	3.741	.83	.07	.64	.04	.00

a. Dependent Variable: SF36BP

Regression SF36GHP

Variables Entered/Removed[a]

Model	Variables Entered	Variables Removed	Method
1	NMDysur, DDysp, NMPPain, NMDysch[b]	.	Enter

a. Dependent Variable: SF36GHP
b. All requested variables entered.

Model Summary

Model	R	R Square	Adjusted R Square	Std. Error of the Estimate
1	.430[a]	.185	.176	21.521

a. Predictors: (Constant), NMDysur, DDysp, NMPPain, NMDysch

ANOVA[a]

Model		Sum of Squares	df	Mean Square	F	Sig.
1	Regression	36710.694	4	9177.674	19.816	<.001[b]
	Residual	161635.735	349	463.140		
	Total	198346.429	353			

a. Dependent Variable: SF36GHP
b. Predictors: (Constant), NMDysur, DDysp, NMPPain, NMDysch

Coefficients[a]

		Unstandardized Coefficients		Standardized Coefficients			Collinearity Statistics	
Model		B	Std. Error	Beta	t	Sig.	Tolerance	VIF
1	(Constant)	26.405	1.975		13.368	<.001		
	DDysp	.788	.319	.128	2.467	.014	.874	1.144
	NMPPain	1.923	.352	.288	5.460	<.001	.842	1.187
	NMDysch	1.342	.452	.157	2.972	.003	.835	1.197
	NMDysur	.041	.637	.003	.064	.949	.917	1.091

a. Dependent Variable: SF36GHP

Bootstrap for Coefficients

Model		B	Bootstrap[a] Bias	Std. Error	Sig. (2-tailed)	95% Confidence Interval Lower	Upper
1	(Constant)	26.405	-.057	1.778	<.001	22.770	29.936
	DDysp	.788	-.001	.323	.018	.157	1.417
	NMPPain	1.923	.015	.340	<.001	1.258	2.599
	NMDysch	1.342	-.011	.500	.006	.366	2.306
	NMDysur	.041	.023	.671	.947	-1.255	1.405

a. Unless otherwise noted, bootstrap results are based on 1000 bootstrap samples

Collinearity Diagnostics[a]

Model	Dimension	Eigenvalue	Condition Index	Variance Proportions (Constant)	DDysp	NMPPain	NMDysch	NMDysur
1	1	3.066	1.000	.03	.03	.03	.04	.02
	2	.839	1.911	.03	.03	.02	.05	.76
	3	.564	2.331	.03	.02	.00	.88	.22
	4	.312	3.135	.07	.85	.31	.00	.00
	5	.219	3.741	.83	.07	.64	.04	.00

a. Dependent Variable: SF36GHP

Regression SF36VIT

Variables Entered/Removed[a]

Model	Variables Entered	Variables Removed	Method
1	NMDysur, DDysp, NMPPain, NMDysch[b]	.	Enter

a. Dependent Variable: SF36VIT
b. All requested variables entered.

Model Summary

Model	R	R Square	Adjusted R Square	Std. Error of the Estimate
1	.419[a]	.176	.166	22.567

a. Predictors: (Constant), NMDysur, DDysp, NMPPain, NMDysch

ANOVA[a]

Model		Sum of Squares	df	Mean Square	F	Sig.
1	Regression	37861.441	4	9465.360	18.586	<.001[b]
	Residual	177733.262	349	509.264		
	Total	215594.703	353			

a. Dependent Variable: SF36VIT
b. Predictors: (Constant), NMDysur, DDysp, NMPPain, NMDysch

Coefficients[a]

		Unstandardized Coefficients		Standardized Coefficients			Collinearity Statistics	
Model		B	Std. Error	Beta	t	Sig.	Tolerance	VIF
1	(Constant)	46.202	2.071		22.307	<.001		
	DDysp	.809	.335	.126	2.417	.016	.874	1.144
	NMPPain	1.653	.369	.237	4.478	<.001	.842	1.187
	NMDysch	1.918	.474	.215	4.050	<.001	.835	1.197
	NMDysur	-.430	.668	-.033	-.644	.520	.917	1.091

a. Dependent Variable: SF36VIT

Bootstrap for Coefficients

Model		B	Bootstrap[a] Bias	Std. Error	Sig. (2-tailed)	95% Confidence Interval Lower	Upper
1	(Constant)	46.202	-.030	1.969	<.001	42.458	50.023
	DDysp	.809	-.008	.338	.018	.119	1.427
	NMPPain	1.653	.000	.390	<.001	.861	2.438
	NMDysch	1.918	-.005	.443	<.001	1.040	2.764
	NMDysur	-.430	.016	.672	.506	-1.732	.914

a. Unless otherwise noted, bootstrap results are based on 1000 bootstrap samples

Collinearity Diagnostics[a]

Model	Dimension	Eigenvalue	Condition Index	Variance Proportions (Constant)	DDysp	NMPPain	NMDysch	NMDysur
1	1	3.066	1.000	.03	.03	.03	.04	.02
	2	.839	1.911	.03	.03	.02	.05	.76
	3	.564	2.331	.03	.02	.00	.88	.22
	4	.312	3.135	.07	.85	.31	.00	.00
	5	.219	3.741	.83	.07	.64	.04	.00

a. Dependent Variable: SF36VIT

Regression SF36SRF

Variables Entered/Removed[a]

Model	Variables Entered	Variables Removed	Method
1	NMDysur, DDysp, NMPPain, NMDysch[b]	.	Enter

a. Dependent Variable: SF36SRF
b. All requested variables entered.

Model Summary

Model	R	R Square	Adjusted R Square	Std. Error of the Estimate
1	.431[a]	.186	.177	26.207

a. Predictors: (Constant), NMDysur, DDysp, NMPPain, NMDysch

ANOVA[a]

Model		Sum of Squares	df	Mean Square	F	Sig.
1	Regression	54774.723	4	13693.681	19.939	<.001[b]
	Residual	239687.639	349	686.784		
	Total	294462.362	353			

a. Dependent Variable: SF36SRF
b. Predictors: (Constant), NMDysur, DDysp, NMPPain, NMDysch

Coefficients[a]

		Unstandardized Coefficients		Standardized Coefficients			Collinearity Statistics	
Model		B	Std. Error	Beta	t	Sig.	Tolerance	VIF
1	(Constant)	27.908	2.405		11.603	<.001		
	DDysp	1.295	.389	.172	3.331	<.001	.874	1.144
	NMPPain	1.564	.429	.192	3.648	<.001	.842	1.187
	NMDysch	2.506	.550	.241	4.556	<.001	.835	1.197
	NMDysur	-.622	.776	-.040	-.801	.423	.917	1.091

a. Dependent Variable: SF36SRF

Bootstrap for Coefficients

Model		B	Bootstrap[a] Bias	Std. Error	Sig. (2-tailed)	95% Confidence Interval Lower	Upper
1	(Constant)	27.908	.050	2.327	<.001	23.352	32.576
	DDysp	1.295	-.009	.394	.002	.453	2.051
	NMPPain	1.564	-.003	.445	<.001	.707	2.501
	NMDysch	2.506	-.031	.531	<.001	1.437	3.467
	NMDysur	-.622	.040	.703	.369	-1.959	.842

a. Unless otherwise noted, bootstrap results are based on 1000 bootstrap samples

Collinearity Diagnostics[a]

Model	Dimension	Eigenvalue	Condition Index	Variance Proportions (Constant)	DDysp	NMPPain	NMDysch	NMDysur
1	1	3.066	1.000	.03	.03	.03	.04	.02
	2	.839	1.911	.03	.03	.02	.05	.76
	3	.564	2.331	.03	.02	.00	.88	.22
	4	.312	3.135	.07	.85	.31	.00	.00
	5	.219	3.741	.83	.07	.64	.04	.00

a. Dependent Variable: SF36SRF

Regression SF36ERF

Variables Entered/Removed[a]

Model	Variables Entered	Variables Removed	Method
1	NMDysur, DDysp, NMPPain, NMDysch[b]	.	Enter

a. Dependent Variable: SF36ERF
b. All requested variables entered.

Model Summary

Model	R	R Square	Adjusted R Square	Std. Error of the Estimate
1	.341[a]	.116	.106	41.284

a. Predictors: (Constant), NMDysur, DDysp, NMPPain, NMDysch

ANOVA[a]

Model		Sum of Squares	df	Mean Square	F	Sig.
1	Regression	78308.019	4	19577.005	11.486	<.001[b]
	Residual	594833.936	349	1704.395		
	Total	673141.955	353			

a. Dependent Variable: SF36ERF
b. Predictors: (Constant), NMDysur, DDysp, NMPPain, NMDysch

Coefficients[a]

		Unstandardized Coefficients		Standardized Coefficients			Collinearity Statistics	
Model		B	Std. Error	Beta	t	Sig.	Tolerance	VIF
1	(Constant)	40.686	3.789		10.738	<.001		
	DDysp	.258	.613	.023	.421	.674	.874	1.144
	NMPPain	2.635	.676	.214	3.900	<.001	.842	1.187
	NMDysch	3.325	.866	.211	3.838	<.001	.835	1.197
	NMDysur	-1.498	1.222	-.064	-1.226	.221	.917	1.091

a. Dependent Variable: SF36ERF

Bootstrap for Coefficients

Model		B	Bootstrap[a] Bias	Std. Error	Sig. (2-tailed)	95% Confidence Interval Lower	Upper
1	(Constant)	40.686	.078	3.777	<.001	33.195	48.068
	DDysp	.258	.011	.627	.707	-.963	1.543
	NMPPain	2.635	-.013	.669	.003	1.225	3.921
	NMDysch	3.325	-.012	.776	<.001	1.778	4.819
	NMDysur	-1.498	.063	1.242	.211	-3.770	1.264

a. Unless otherwise noted, bootstrap results are based on 1000 bootstrap samples

Collinearity Diagnostics[a]

Model	Dimension	Eigenvalue	Condition Index	Variance Proportions (Constant)	DDysp	NMPPain	NMDysch	NMDysur
1	1	3.066	1.000	.03	.03	.03	.04	.02
	2	.839	1.911	.03	.03	.02	.05	.76
	3	.564	2.331	.03	.02	.00	.88	.22
	4	.312	3.135	.07	.85	.31	.00	.00
	5	.219	3.741	.83	.07	.64	.04	.00

a. Dependent Variable: SF36ERF

Regression SF36MH

Variables Entered/Removed[a]

Model	Variables Entered	Variables Removed	Method
1	NMDysur, DDysp, NMPPain, NMDysch[b]	.	Enter

a. Dependent Variable: SF36MH
b. All requested variables entered.

Model Summary

Model	R	R Square	Adjusted R Square	Std. Error of the Estimate
1	.409[a]	.167	.158	20.241

a. Predictors: (Constant), NMDysur, DDysp, NMPPain, NMDysch

ANOVA[a]

Model		Sum of Squares	df	Mean Square	F	Sig.
1	Regression	28762.742	4	7190.686	17.551	<.001[b]
	Residual	142988.535	349	409.709		
	Total	171751.277	353			

a. Dependent Variable: SF36MH
b. Predictors: (Constant), NMDysur, DDysp, NMPPain, NMDysch

Coefficients[a]

		Unstandardized Coefficients		Standardized Coefficients			Collinearity Statistics	
Model		B	Std. Error	Beta	t	Sig.	Tolerance	VIF
1	(Constant)	34.820	1.858		18.743	<.001		
	DDysp	.470	.300	.082	1.564	.119	.874	1.144
	NMPPain	1.747	.331	.281	5.275	<.001	.842	1.187
	NMDysch	1.499	.425	.189	3.528	<.001	.835	1.197
	NMDysur	-.595	.599	-.051	-.994	.321	.917	1.091

a. Dependent Variable: SF36MH

Bootstrap for Coefficients

Model		B	Bootstrap[a] Bias	Std. Error	Sig. (2-tailed)	95% Confidence Interval Lower	Upper
1	(Constant)	34.820	-.023	1.731	<.001	31.506	38.420
	DDysp	.470	.001	.296	.118	-.124	1.028
	NMPPain	1.747	.013	.338	<.001	1.118	2.448
	NMDysch	1.499	-.019	.434	.002	.652	2.280
	NMDysur	-.595	-.014	.561	.267	-1.702	.483

a. Unless otherwise noted, bootstrap results are based on 1000 bootstrap samples

Collinearity Diagnostics[a]

Model	Dimension	Eigenvalue	Condition Index	Variance Proportions (Constant)	DDysp	NMPPain	NMDysch	NMDysur
1	1	3.066	1.000	.03	.03	.03	.04	.02
	2	.839	1.911	.03	.03	.02	.05	.76
	3	.564	2.331	.03	.02	.00	.88	.22
	4	.312	3.135	.07	.85	.31	.00	.00
	5	.219	3.741	.83	.07	.64	.04	.00

a. Dependent Variable: SF36MH

Regression EHP30Pain

Variables Entered/Removed[a]

Model	Variables Entered	Variables Removed	Method
1	NMDysur, DDysp, NMDysch, NMPPain[b]	.	Enter

a. Dependent Variable: EHP30Pain
b. All requested variables entered.

Model Summary

Model	R	R Square	Adjusted R Square	Std. Error of the Estimate
1	.505[a]	.255	.244	23.674

a. Predictors: (Constant), NMDysur, DDysp, NMDysch, NMPPain

ANOVA[a]

Model		Sum of Squares	df	Mean Square	F	Sig.
1	Regression	50991.918	4	12747.979	22.746	<.001[b]
	Residual	149080.525	266	560.453		
	Total	200072.443	270			

a. Dependent Variable: EHP30Pain
b. Predictors: (Constant), NMDysur, DDysp, NMDysch, NMPPain

Coefficients[a]

		Unstandardized Coefficients		Standardized Coefficients			Collinearity Statistics	
Model		B	Std. Error	Beta	t	Sig.	Tolerance	VIF
1	(Constant)	21.004	2.493		8.427	<.001		
	DDysp	.779	.407	.109	1.913	.057	.869	1.151
	NMPPain	2.557	.450	.331	5.676	<.001	.823	1.216
	NMDysch	2.411	.578	.237	4.168	<.001	.866	1.155
	NMDysur	-.486	.820	-.032	-.593	.554	.938	1.066

a. Dependent Variable: EHP30Pain

Bootstrap for Coefficients

Model		B	Bootstrap[a] Bias	Std. Error	Sig. (2-tailed)	95% Confidence Interval Lower	Upper
1	(Constant)	21.004	-.027	2.372	<.001	16.413	25.858
	DDysp	.779	-.023	.424	.068	-.087	1.556
	NMPPain	2.557	.015	.485	<.001	1.618	3.548
	NMDysch	2.411	-.007	.576	<.001	1.270	3.585
	NMDysur	-.486	.022	.761	.497	-1.955	1.075

a. Unless otherwise noted, bootstrap results are based on 1000 bootstrap samples

Collinearity Diagnostics[a]

Model	Dimension	Eigenvalue	Condition Index	Variance Proportions (Constant)	DDysp	NMPPain	NMDysch	NMDysur
1	1	3.048	1.000	.03	.03	.03	.04	.02
	2	.833	1.913	.03	.02	.01	.02	.86
	3	.603	2.247	.03	.03	.01	.91	.11
	4	.301	3.182	.12	.89	.25	.00	.00
	5	.215	3.763	.79	.03	.70	.03	.00

a. Dependent Variable: EHP30Pain

Regression EHP30CPower

Variables Entered/Removed[a]

Model	Variables Entered	Variables Removed	Method
1	NMDysur, DDysp, NMDysch, NMPPain[b]	.	Enter

a. Dependent Variable: EHP30CPower
b. All requested variables entered.

Model Summary

Model	R	R Square	Adjusted R Square	Std. Error of the Estimate
1	.511[a]	.262	.250	27.303

a. Predictors: (Constant), NMDysur, DDysp, NMDysch, NMPPain

ANOVA[a]

Model		Sum of Squares	df	Mean Square	F	Sig.
1	Regression	70252.206	4	17563.052	23.559	<.001[b]
	Residual	198297.181	266	745.478		
	Total	268549.387	270			

a. Dependent Variable: EHP30CPower
b. Predictors: (Constant), NMDysur, DDysp, NMDysch, NMPPain

Coefficients[a]

		Unstandardized Coefficients		Standardized Coefficients			Collinearity Statistics	
Model		B	Std. Error	Beta	t	Sig.	Tolerance	VIF
1	(Constant)	30.364	2.875		10.562	<.001		
	DDysp	.964	.470	.116	2.053	.041	.869	1.151
	NMPPain	3.194	.520	.357	6.148	<.001	.823	1.216
	NMDysch	2.488	.667	.211	3.729	<.001	.866	1.155
	NMDysur	-.803	.946	-.046	-.849	.397	.938	1.066

a. Dependent Variable: EHP30CPower

Bootstrap for Coefficients

Model		B	Bootstrap[a] Bias	Std. Error	Sig. (2-tailed)	95% Confidence Interval Lower	Upper
1	(Constant)	30.364	-.266	2.856	<.001	24.581	35.859
	DDysp	.964	.025	.463	.034	.085	1.853
	NMPPain	3.194	.012	.523	<.001	2.146	4.219
	NMDysch	2.488	.029	.556	<.001	1.308	3.582
	NMDysur	-.803	-.020	.946	.399	-2.598	.996

a. Unless otherwise noted, bootstrap results are based on 1000 bootstrap samples

Collinearity Diagnostics[a]

Model	Dimension	Eigenvalue	Condition Index	Variance Proportions (Constant)	DDysp	NMPPain	NMDysch	NMDysur
1	1	3.048	1.000	.03	.03	.03	.04	.02
	2	.833	1.913	.03	.02	.01	.02	.86
	3	.603	2.247	.03	.03	.01	.91	.11
	4	.301	3.182	.12	.89	.25	.00	.00
	5	.215	3.763	.79	.03	.70	.03	.00

a. Dependent Variable: EHP30CPower

Regression EHP30SSupp

Variables Entered/Removed[a]

Model	Variables Entered	Variables Removed	Method
1	NMDysur, DDysp, NMDysch, NMPPain[b]	.	Enter

a. Dependent Variable: EHP30SSupp
b. All requested variables entered.

Model Summary

Model	R	R Square	Adjusted R Square	Std. Error of the Estimate
1	.449[a]	.202	.190	26.590

a. Predictors: (Constant), NMDysur, DDysp, NMDysch, NMPPain

ANOVA[a]

Model		Sum of Squares	df	Mean Square	F	Sig.
1	Regression	47597.122	4	11899.280	16.830	<.001[b]
	Residual	188068.974	266	707.026		
	Total	235666.096	270			

a. Dependent Variable: EHP30SSupp
b. Predictors: (Constant), NMDysur, DDysp, NMDysch, NMPPain

Coefficients[a]

		Unstandardized Coefficients		Standardized Coefficients			Collinearity Statistics	
Model		B	Std. Error	Beta	t	Sig.	Tolerance	VIF
1	(Constant)	27.672	2.800		9.884	<.001		
	DDysp	.919	.457	.118	2.010	.045	.869	1.151
	NMPPain	2.589	.506	.309	5.117	<.001	.823	1.216
	NMDysch	2.019	.650	.183	3.108	.002	.866	1.155
	NMDysur	-1.050	.921	-.064	-1.140	.255	.938	1.066

a. Dependent Variable: EHP30SSupp

Bootstrap for Coefficients

Model		B	Bootstrap[a] Bias	Std. Error	Sig. (2-tailed)	95% Confidence Interval Lower	Upper
1	(Constant)	27.672	.043	2.776	<.001	22.377	33.244
	DDysp	.919	-.008	.472	.058	-.023	1.876
	NMPPain	2.589	.018	.540	<.001	1.565	3.658
	NMDysch	2.019	.015	.664	.003	.744	3.375
	NMDysur	-1.050	-.040	.832	.203	-2.850	.466

a. Unless otherwise noted, bootstrap results are based on 1000 bootstrap samples

Collinearity Diagnostics[a]

Model	Dimension	Eigenvalue	Condition Index	Variance Proportions (Constant)	DDysp	NMPPain	NMDysch	NMDysur
1	1	3.048	1.000	.03	.03	.03	.04	.02
	2	.833	1.913	.03	.02	.01	.02	.86
	3	.603	2.247	.03	.03	.01	.91	.11
	4	.301	3.182	.12	.89	.25	.00	.00
	5	.215	3.763	.79	.03	.70	.03	.00

a. Dependent Variable: EHP30SSupp

Regression EHP30EWbeing

Variables Entered/Removed[a]

Model	Variables Entered	Variables Removed	Method
1	NMDysur, DDysp, NMDysch, NMPPain[b]	.	Enter

a. Dependent Variable: EHP30EWbeing
b. All requested variables entered.

Model Summary

Model	R	R Square	Adjusted R Square	Std. Error of the Estimate
1	.437[a]	.191	.179	22.953

a. Predictors: (Constant), NMDysur, DDysp, NMDysch, NMPPain

ANOVA[a]

Model		Sum of Squares	df	Mean Square	F	Sig.
1	Regression	33142.912	4	8285.728	15.728	<.001[b]
	Residual	140133.434	266	526.817		
	Total	173276.347	270			

a. Dependent Variable: EHP30EWbeing
b. Predictors: (Constant), NMDysur, DDysp, NMDysch, NMPPain

Coefficients[a]

Model		Unstandardized Coefficients		Standardized Coefficients			Collinearity Statistics	
		B	Std. Error	Beta	t	Sig.	Tolerance	VIF
1	(Constant)	34.159	2.417		14.135	<.001		
	DDysp	.485	.395	.073	1.228	.220	.869	1.151
	NMPPain	2.191	.437	.305	5.017	<.001	.823	1.216
	NMDysch	1.976	.561	.209	3.523	<.001	.866	1.155
	NMDysur	-1.465	.795	-.105	-1.843	.066	.938	1.066

a. Dependent Variable: EHP30EWbeing

Bootstrap for Coefficients

Model		B	Bootstrap[a] Bias	Std. Error	Sig. (2-tailed)	95% Confidence Interval Lower	Upper
1	(Constant)	34.159	-.130	2.406	<.001	29.274	38.916
	DDysp	.485	.019	.402	.239	-.269	1.306
	NMPPain	2.191	.010	.466	<.001	1.298	3.149
	NMDysch	1.976	.017	.541	<.001	.914	3.134
	NMDysur	-1.465	-.023	.691	.031	-2.913	-.200

a. Unless otherwise noted, bootstrap results are based on 1000 bootstrap samples

Collinearity Diagnostics[a]

Model	Dimension	Eigenvalue	Condition Index	Variance Proportions (Constant)	DDysp	NMPPain	NMDysch	NMDysur
1	1	3.048	1.000	.03	.03	.03	.04	.02
	2	.833	1.913	.03	.02	.01	.02	.86
	3	.603	2.247	.03	.03	.01	.91	.11
	4	.301	3.182	.12	.89	.25	.00	.00
	5	.215	3.763	.79	.03	.70	.03	.00

a. Dependent Variable: EHP30EWbeing

Regression EHP30SImage

Variables Entered/Removed[a]

Model	Variables Entered	Variables Removed	Method
1	NMDysur, DDysp, NMDysch, NMPPain[b]	.	Enter

a. Dependent Variable: EHP30SImage
b. All requested variables entered.

Model Summary

Model	R	R Square	Adjusted R Square	Std. Error of the Estimate
1	.391[a]	.153	.140	29.261

a. Predictors: (Constant), NMDysur, DDysp, NMDysch, NMPPain

ANOVA[a]

Model		Sum of Squares	df	Mean Square	F	Sig.
1	Regression	41172.775	4	10293.194	12.022	<.001[b]
	Residual	227756.864	266	856.229		
	Total	268929.638	270			

a. Dependent Variable: EHP30SImage
b. Predictors: (Constant), NMDysur, DDysp, NMDysch, NMPPain

Coefficients[a]

		Unstandardized Coefficients		Standardized Coefficients			Collinearity Statistics	
Model		B	Std. Error	Beta	t	Sig.	Tolerance	VIF
1	(Constant)	27.347	3.081		8.877	<.001		
	DDysp	.497	.503	.060	.987	.325	.869	1.151
	NMPPain	2.526	.557	.282	4.537	<.001	.823	1.216
	NMDysch	1.988	.715	.169	2.781	.006	.866	1.155
	NMDysur	-2.843	1.013	-.163	-2.806	.005	.938	1.066

a. Dependent Variable: EHP30SImage

Bootstrap for Coefficients

Model		B	Bootstrap[a] Bias	Std. Error	Sig. (2-tailed)	95% Confidence Interval Lower	Upper
1	(Constant)	27.347	-.106	2.846	<.001	21.452	32.979
	DDysp	.497	-.003	.517	.340	-.552	1.567
	NMPPain	2.526	.011	.592	<.001	1.394	3.710
	NMDysch	1.988	.035	.707	.006	.590	3.370
	NMDysur	-2.843	-.030	.992	.005	-5.004	-.900

a. Unless otherwise noted, bootstrap results are based on 1000 bootstrap samples

Collinearity Diagnostics[a]

Model	Dimension	Eigenvalue	Condition Index	Variance Proportions (Constant)	DDysp	NMPPain	NMDysch	NMDysur
1	1	3.048	1.000	.03	.03	.03	.04	.02
	2	.833	1.913	.03	.02	.01	.02	.86
	3	.603	2.247	.03	.03	.01	.91	.11
	4	.301	3.182	.12	.89	.25	.00	.00
	5	.215	3.763	.79	.03	.70	.03	.00

a. Dependent Variable: EHP30SImage

Regression EHP30Sex

Variables Entered/Removed[a]

Model	Variables Entered	Variables Removed	Method
1	NMDysur, DDysp, NMDysch, NMPPain[b]	.	Enter

a. Dependent Variable: EHP30Sex
b. All requested variables entered.

Model Summary

Model	R	R Square	Adjusted R Square	Std. Error of the Estimate
1	.651[a]	.424	.414	24.913

a. Predictors: (Constant), NMDysur, DDysp, NMDysch, NMPPain

ANOVA[a]

Model		Sum of Squares	df	Mean Square	F	Sig.
1	Regression	113106.062	4	28276.516	45.559	<.001[b]
	Residual	153924.570	248	620.664		
	Total	267030.632	252			

a. Dependent Variable: EHP30Sex
b. Predictors: (Constant), NMDysur, DDysp, NMDysch, NMPPain

Coefficients[a]

		Unstandardized Coefficients		Standardized Coefficients			Collinearity Statistics	
Model		B	Std. Error	Beta	t	Sig.	Tolerance	VIF
1	(Constant)	18.136	2.756		6.582	<.001		
	DDysp	4.903	.444	.571	11.047	<.001	.871	1.148
	NMPPain	1.341	.488	.146	2.748	.006	.825	1.213
	NMDysch	.434	.627	.036	.692	.490	.856	1.168
	NMDysur	.568	.865	.033	.656	.512	.938	1.066

a. Dependent Variable: EHP30Sex

Bootstrap for Coefficients

Model		B	Bootstrap[a] Bias	Std. Error	Sig. (2-tailed)	95% Confidence Interval Lower	Upper
1	(Constant)	18.136	-.143	2.259	<.001	13.544	22.385
	DDysp	4.903	.015	.459	<.001	4.023	5.824
	NMPPain	1.341	.010	.510	.012	.348	2.348
	NMDysch	.434	-.009	.594	.460	-.732	1.591
	NMDysur	.568	-.037	.937	.534	-1.325	2.392

a. Unless otherwise noted, bootstrap results are based on 1000 bootstrap samples

Collinearity Diagnostics[a]

Model	Dimension	Eigenvalue	Condition Index	Variance Proportions (Constant)	DDysp	NMPPain	NMDysch	NMDysur
1	1	3.076	1.000	.03	.03	.03	.04	.02
	2	.825	1.931	.03	.02	.01	.01	.87
	3	.595	2.274	.04	.03	.01	.91	.11
	4	.295	3.230	.10	.87	.29	.00	.00
	5	.209	3.835	.81	.05	.67	.04	.00

a. Dependent Variable: EHP30Sex

Regression EHP30Work

Variables Entered/Removed[a]

Model	Variables Entered	Variables Removed	Method
1	NMDysur, DDysp, NMDysch, NMPPain[b]	.	Enter

a. Dependent Variable: EHP30Work
b. All requested variables entered.

Model Summary

Model	R	R Square	Adjusted R Square	Std. Error of the Estimate
1	.453[a]	.206	.190	25.688

a. Predictors: (Constant), NMDysur, DDysp, NMDysch, NMPPain

ANOVA[a]

Model		Sum of Squares	df	Mean Square	F	Sig.
1	Regression	34843.671	4	8710.918	13.201	<.001[b]
	Residual	134610.157	204	659.854		
	Total	169453.828	208			

a. Dependent Variable: EHP30Work
b. Predictors: (Constant), NMDysur, DDysp, NMDysch, NMPPain

Coefficients[a]

		Unstandardized Coefficients		Standardized Coefficients			Collinearity Statistics	
Model		B	Std. Error	Beta	t	Sig.	Tolerance	VIF
1	(Constant)	14.283	3.046		4.689	<.001		
	DDysp	1.207	.508	.158	2.375	.018	.881	1.135
	NMPPain	2.303	.559	.285	4.121	<.001	.816	1.225
	NMDysch	1.826	.728	.168	2.508	.013	.866	1.154
	NMDysur	-.116	1.098	-.007	-.106	.916	.967	1.034

a. Dependent Variable: EHP30Work

Bootstrap for Coefficients

Model		B	Bootstrap[a] Bias	Std. Error	Sig. (2-tailed)	95% Confidence Interval Lower	Upper
1	(Constant)	14.283	-.073	2.606	<.001	9.304	19.470
	DDysp	1.207	.000	.526	.023	.201	2.258
	NMPPain	2.303	.008	.606	<.001	1.097	3.485
	NMDysch	1.826	-.013	.711	.009	.369	3.171
	NMDysur	-.116	.042	1.091	.922	-2.243	2.171

a. Unless otherwise noted, bootstrap results are based on 1000 bootstrap samples

Collinearity Diagnostics[a]

Model	Dimension	Eigenvalue	Condition Index	Variance Proportions (Constant)	DDysp	NMPPain	NMDysch	NMDysur
1	1	2.972	1.000	.03	.03	.03	.04	.02
	2	.869	1.849	.01	.02	.01	.00	.94
	3	.633	2.167	.05	.04	.01	.90	.04
	4	.304	3.128	.11	.86	.30	.01	.01
	5	.222	3.661	.80	.05	.66	.05	.00

a. Dependent Variable: EHP30Work

Regression EHP30FMed

Variables Entered/Removed[a]

Model	Variables Entered	Variables Removed	Method
1	NMDysur, DDysp, NMDysch, NMPPain[b]	.	Enter

a. Dependent Variable: EHP30FMed
b. All requested variables entered.

Model Summary

Model	R	R Square	Adjusted R Square	Std. Error of the Estimate
1	.176[a]	.031	.016	30.253

a. Predictors: (Constant), NMDysur, DDysp, NMDysch, NMPPain

ANOVA[a]

Model		Sum of Squares	df	Mean Square	F	Sig.
1	Regression	7296.247	4	1824.062	1.993	.096[b]
	Residual	226975.650	248	915.224		
	Total	234271.897	252			

a. Dependent Variable: EHP30FMed
b. Predictors: (Constant), NMDysur, DDysp, NMDysch, NMPPain

Coefficients[a]

		Unstandardized Coefficients		Standardized Coefficients			Collinearity Statistics	
Model		B	Std. Error	Beta	t	Sig.	Tolerance	VIF
1	(Constant)	17.199	3.277		5.248	<.001		
	DDysp	.482	.549	.060	.877	.381	.847	1.180
	NMPPain	.463	.607	.053	.762	.447	.798	1.253
	NMDysch	1.439	.757	.128	1.901	.058	.864	1.158
	NMDysur	-1.118	1.065	-.068	-1.050	.295	.933	1.071

a. Dependent Variable: EHP30FMed

Bootstrap for Coefficients

Model		B	Bootstrap[a] Bias	Std. Error	Sig. (2-tailed)	95% Confidence Interval Lower	Upper
1	(Constant)	17.199	-.174	3.131	<.001	11.377	23.911
	DDysp	.482	.016	.518	.351	-.510	1.523
	NMPPain	.463	.012	.543	.393	-.593	1.571
	NMDysch	1.439	.023	.880	.096	-.228	3.286
	NMDysur	-1.118	.003	1.187	.309	-3.354	1.527

a. Unless otherwise noted, bootstrap results are based on 1000 bootstrap samples

Collinearity Diagnostics[a]

Model	Dimension	Eigenvalue	Condition Index	Variance Proportions (Constant)	DDysp	NMPPain	NMDysch	NMDysur
1	1	3.075	1.000	.03	.03	.03	.04	.02
	2	.830	1.925	.03	.02	.01	.02	.85
	3	.593	2.277	.03	.03	.01	.91	.12
	4	.285	3.286	.19	.91	.20	.00	.00
	5	.217	3.762	.72	.00	.76	.03	.00

a. Dependent Variable: EHP30FMed

Regression EHP30Infert

Variables Entered/Removed[a]

Model	Variables Entered	Variables Removed	Method
1	NMDysur, DDysp, NMDysch, NMPPain[b]	.	Enter

a. Dependent Variable: EHP30Infert
b. All requested variables entered.

Model Summary

Model	R	R Square	Adjusted R Square	Std. Error of the Estimate
1	.164[a]	.027	.008	30.490

a. Predictors: (Constant), NMDysur, DDysp, NMDysch, NMPPain

ANOVA[a]

Model		Sum of Squares	df	Mean Square	F	Sig.
1	Regression	5372.414	4	1343.104	1.445	.220[b]
	Residual	195225.325	210	929.644		
	Total	200597.740	214			

a. Dependent Variable: EHP30Infert
b. Predictors: (Constant), NMDysur, DDysp, NMDysch, NMPPain

Coefficients[a]

		Unstandardized Coefficients		Standardized Coefficients			Collinearity Statistics	
Model		B	Std. Error	Beta	t	Sig.	Tolerance	VIF
1	(Constant)	49.434	3.595		13.752	<.001		
	DDysp	.756	.595	.093	1.271	.205	.859	1.164
	NMPPain	.218	.660	.025	.330	.742	.808	1.237
	NMDysch	.697	.845	.060	.825	.410	.879	1.138
	NMDysur	-2.099	1.177	-.125	-1.784	.076	.947	1.056

a. Dependent Variable: EHP30Infert

Bootstrap for Coefficients

Model		B	Bootstrap[a] Bias	Std. Error	Sig. (2-tailed)	95% Confidence Interval Lower	Upper
1	(Constant)	49.434	.045	3.561	<.001	42.434	56.932
	DDysp	.756	-.015	.571	.183	-.407	1.869
	NMPPain	.218	-.024	.627	.715	-1.046	1.455
	NMDysch	.697	.042	.900	.450	-1.070	2.435
	NMDysur	-2.099	-.020	1.122	.047	-4.228	.095

a. Unless otherwise noted, bootstrap results are based on 1000 bootstrap samples

Collinearity Diagnostics[a]

Model	Dimension	Eigenvalue	Condition Index	Variance Proportions (Constant)	DDysp	NMPPain	NMDysch	NMDysur
1	1	3.039	1.000	.03	.03	.03	.04	.02
	2	.830	1.914	.02	.02	.01	.00	.92
	3	.630	2.197	.04	.03	.01	.92	.05
	4	.278	3.309	.07	.83	.40	.01	.00
	5	.224	3.685	.84	.09	.56	.03	.01

a. Dependent Variable: EHP30Infert

Regression EHP30RChild

Warnings

For models with dependent variable EHP30RChild, the following variables are constants or have missing correlations in split file $bootstrap_split=78 : NMDysur. They will be deleted from the analysis.
For models with dependent variable EHP30RChild, the following variables are constants or have missing correlations in split file $bootstrap_split=153 : NMDysur. They will be deleted from the analysis.
For models with dependent variable EHP30RChild, the following variables are constants or have missing correlations in split file $bootstrap_split=217 : NMDysur. They will be deleted from the analysis.
For models with dependent variable EHP30RChild, the following variables are constants or have missing correlations in split file $bootstrap_split=285 : NMDysur. They will be deleted from the analysis.
For models with dependent variable EHP30RChild, the following variables are constants or have missing correlations in split file $bootstrap_split=557 : NMDysur. They will be deleted from the analysis.
For models with dependent variable EHP30RChild, the following variables are constants or have missing correlations in split file $bootstrap_split=595 : NMDysur. They will be deleted from the analysis.
For models with dependent variable EHP30RChild, the following variables are constants or have missing correlations in split file $bootstrap_split=713 : NMDysur. They will be deleted from the analysis.
For models with dependent variable EHP30RChild, the following variables are constants or have missing correlations in split file $bootstrap_split=747 : NMDysur. They will be deleted from the analysis.
For models with dependent variable EHP30RChild, the following variables are constants or have missing correlations in split file $bootstrap_split=797 : NMDysur. They will be deleted from the analysis.
For models with dependent variable EHP30RChild, the following variables are constants or have missing correlations in split file $bootstrap_split=945 : NMDysur. They will be deleted from the analysis.
For models with dependent variable EHP30RChild, the following variables are constants or have missing correlations in split file $bootstrap_split=984 : NMDysur. They will be deleted from the analysis.

Variables Entered/Removed[a]

Model	Variables Entered	Variables Removed	Method
1	NMDysur, NMPPain, NMDysch, DDysp[b]	.	Enter

a. Dependent Variable: EHP30RChild
b. All requested variables entered.

Model Summary

Model	R	R Square	Adjusted R Square	Std. Error of the Estimate
1	.375[a]	.140	.088	29.922

a. Predictors: (Constant), NMDysur, NMPPain, NMDysch, DDysp

ANOVA[a]

Model		Sum of Squares	df	Mean Square	F	Sig.
1	Regression	9644.712	4	2411.178	2.693	.038[b]
	Residual	59090.217	66	895.306		
	Total	68734.930	70			

a. Dependent Variable: EHP30RChild
b. Predictors: (Constant), NMDysur, NMPPain, NMDysch, DDysp

Coefficients[a]

Model		Unstandardized Coefficients		Standardized Coefficients			Collinearity Statistics	
		B	Std. Error	Beta	t	Sig.	Tolerance	VIF
1	(Constant)	22.150	6.125		3.616	<.001		
	DDysp	-.572	1.096	-.074	-.522	.603	.655	1.528
	NMPPain	2.890	1.165	.340	2.480	.016	.693	1.442
	NMDysch	1.459	1.537	.123	.949	.346	.771	1.297
	NMDysur	1.012	2.647	.045	.382	.703	.923	1.083

a. Dependent Variable: EHP30RChild

Bootstrap for Coefficients

Model		B	Bootstrap[a]				
			Bias	Std. Error	Sig. (2-tailed)	95% Confidence Interval Lower	Upper
1	(Constant)	22.150	-.087[b]	5.443[b]	.001[b]	11.922[b]	33.481[b]
	DDysp	-.572	.063[b]	1.093[b]	.629[b]	-2.762[b]	1.611[b]
	NMPPain	2.890	.053[b]	1.096[b]	.016[b]	.645[b]	5.029[b]
	NMDysch	1.459	-.244[b]	1.897[b]	.403[b]	-2.981[b]	4.536[b]
	NMDysur	1.012	-2.991[b]	7.299[b]	.599[b]	-23.643[b]	4.092[b]

a. Unless otherwise noted, bootstrap results are based on 1000 bootstrap samples

b. Based on 989 samples

Collinearity Diagnostics[a]

Model	Dimension	Eigenvalue	Condition Index	Variance Proportions				
				(Constant)	DDysp	NMPPain	NMDysch	NMDysur
1	1	3.082	1.000	.03	.03	.02	.04	.02
	2	.917	1.833	.03	.00	.01	.02	.81
	3	.553	2.360	.08	.00	.01	.80	.17
	4	.283	3.299	.37	.76	.00	.13	.00
	5	.164	4.335	.50	.21	.95	.01	.00

a. Dependent Variable: EHP30RChild

Regression EHP30FTreat

Variables Entered/Removed[a]

Model	Variables Entered	Variables Removed	Method
1	NMDysur, DDysp, NMDysch, NMPPain[b]	.	Enter

a. Dependent Variable: EHP30FTreat
b. All requested variables entered.

Model Summary

Model	R	R Square	Adjusted R Square	Std. Error of the Estimate
1	.336[a]	.113	.094	28.596

a. Predictors: (Constant), NMDysur, DDysp, NMDysch, NMPPain

ANOVA[a]

Model		Sum of Squares	df	Mean Square	F	Sig.
1	Regression	20035.848	4	5008.962	6.125	<.001[b]
	Residual	157825.693	193	817.750		
	Total	177861.540	197			

a. Dependent Variable: EHP30FTreat
b. Predictors: (Constant), NMDysur, DDysp, NMDysch, NMPPain

Coefficients[a]

		Unstandardized Coefficients		Standardized Coefficients			Collinearity Statistics	
Model		B	Std. Error	Beta	t	Sig.	Tolerance	VIF
1	(Constant)	35.368	3.497		10.114	<.001		
	DDysp	-.252	.563	-.033	-.447	.655	.860	1.163
	NMPPain	2.635	.633	.316	4.161	<.001	.795	1.258
	NMDysch	1.001	.796	.092	1.257	.210	.864	1.157
	NMDysur	-.873	1.204	-.051	-.725	.469	.919	1.088

a. Dependent Variable: EHP30FTreat

Bootstrap for Coefficients

Model		B	Bootstrap[a] Bias	Std. Error	Sig. (2-tailed)	95% Confidence Interval Lower	Upper
1	(Constant)	35.368	.019	3.759	<.001	27.186	42.946
	DDysp	-.252	-.035	.502	.586	-1.285	.733
	NMPPain	2.635	.034	.573	<.001	1.474	3.767
	NMDysch	1.001	.024	.812	.204	-.623	2.601
	NMDysur	-.873	.086	1.617	.601	-3.944	2.586

a. Unless otherwise noted, bootstrap results are based on 1000 bootstrap samples

Collinearity Diagnostics[a]

Model	Dimension	Eigenvalue	Condition Index	Variance Proportions (Constant)	DDysp	NMPPain	NMDysch	NMDysur
1	1	3.071	1.000	.03	.03	.03	.04	.02
	2	.844	1.907	.03	.03	.01	.03	.80
	3	.575	2.310	.03	.03	.01	.91	.16
	4	.292	3.241	.21	.90	.18	.01	.00
	5	.217	3.764	.71	.00	.78	.02	.01

a. Dependent Variable: EHP30FTreat
