# Supplementary material for: Non-menstrual pelvic symptoms and women’s quality of life: a cross-sectional observational study
Source: PLoS One. 2025 Apr 29;20(4):e0321922. doi: 10.1371/journal.pone.0321922 (PMC12040126; doi:10.1371/journal.pone.0321922)
Supplement: Attachment — Submitted filename: [2] Response to Reviewers_PONE.docx [file pone.0321922.s003.docx]

August 21, 2024

Dear Editor in Chief,

The co-authors and I are resubmitting the manuscript “THE BURDEN OF DIFFERENT ENDOMETRIOSIS-RELATED NON-MENSTRUAL PELVIC SYMPTOMS ON WOMEN'S QUALITY OF LIFE: AN EXPLORATORY OBSERVATIONAL STUDY" for your consideration.

There were prior interactions with PLOS ONE regarding this manuscript (PONE-D-24-25332). Following specific guidance that we received from the PLOS ONE Editorial Office on 3/July/2024, we have uploaded this specific response as a “Response to Reviewers” file type.

At this time, I would like to sincerely request that I be offered an opportunity to clarify any queries so that our article can be considered for publication.

*PLOS ONE Decision: PONE-D-24-25332 - [EMID:0e6bd00b7df774fe]:*

*“…We recognize that you obtained ethical approval from your local ethics committee after you began the study: your ethics approval document is dated 21 Feb 2019, while participants' data were accessed from 01 Jan 2018 to 30 July 2021. For human subjects research, PLOS ONE requires that authors obtain ethics committee approval before beginning the study, and we do not accept retrospective ethical approval.”*

As described in the METHODS section, this is a retrospective study, that is, we correctly obtained approval to use data already existing in medical records (retrospective data). Unfortunately, we realize that we may not have been clear about such an important issue in the first submission. Maybe, we presented too many (unnecessary) details, which ended up causing confusion.

We hope it is now clear that our institutional review board approved a retrospective observational study, which meets all applicable standards for the ethics of experimentation and research integrity.

Thank you very much for your attention.

Yours sincerely,

Marlon de Freitas Fonseca

Brazil

Corresponding author’s e-mail: [marlon.iff@gmail.com](mailto:marlon.iff@gmail.com)

Tel: +55 21 99631-9500
