# Supplementary material for: Non-menstrual pelvic symptoms and women’s quality of life: a cross-sectional observational study
Source: PLoS One. 2025 Apr 29;20(4):e0321922. doi: 10.1371/journal.pone.0321922 (PMC12040126; doi:10.1371/journal.pone.0321922)
Supplement: Attachment — Submitted filename: Response to Reviewers.docx [file pone.0321922.s004.docx]

Journal Requirements:

*Dear Reviewers,*

*Dear Editor,*

*We would like to thank you for this opportunity.*

*This study has undoubtedly been greatly improved thanks to the suggestions we have received.*

*As already mentioned, this manuscript was written for readers from different areas. Therefore, we have had a challenge: to present details (statistical rigor to obtain the results), while at the same time providing an easy and understandable reading of the discussion and conclusion.*

*Changes have been made to some abbreviations, some expressions and some mathematical details. A supplementary PDF file has been added. Some paragraphs have been rewritten to make them easier to read. A total of 10 references have been added to address reviewers' comments and suggestions.*

*We hope we have succeeded.*

*Thank you.*

*marlon & Col.*

1. Please ensure that your manuscript meets PLOS ONE's style requirements, including those for file naming. The PLOS ONE style templates can be found at

<https://journals.plos.org/plosone/s/file?id=wjVg/PLOSOne_formatting_sample_main_body.pdf> and [https://journals.plos.org/plosone/s/file?id=ba62/PLOSOne_formatting_sample_title_authors_affiliations.pdf](https://track.editorialmanager.com/CL0/https:%2F%2Fjournals.plos.org%2Fplosone%2Fs%2Ffile%3Fid=ba62%2FPLOSOne_formatting_sample_title_authors_affiliations.pdf/1/010f01952a9cd336-271871ee-fc2b-48e0-9bde-f6602fdbc31e-000000/BXROhvoLUoQcNeRrM-mdjNlkMzW5JKtDFD67aHoLppg=199)

AUTHORES REPLY: Corrections were made according to PLOS ONE's style requirements. Thank you!

2.    We noticed you have some minor occurrence of overlapping text with the following previous publication(s), which needs to be addressed:

-<https://pure.uvt.nl/ws/portalfiles/portal/266549/whoqolgroupbref1998.pdf>

- <https://doi.org/10.3390/ijtm2040041>

In your revision ensure you cite all your sources (including your own works), and quote or rephrase any duplicated text outside the methods section. Further consideration is dependent on these concerns being addressed.

AUTHORES REPLY: It was corrected.

-<https://pure.uvt.nl/ws/portalfiles/portal/266549/whoqolgroupbref1998.pdf>

This publication was already in the reference list. It was correctly positioned in the manuscript. Please see 2^nd^ paragraph of INTRODUCTION Section (below).

“*Potentially influenced by biological, psychological and social factors, pain may be defined as an unpleasant sensory and emotional experience associated with, or resembling that associated with, actual or potential tissue damage [7]. Quality of life may be defined as individuals’ perceptions of their position in life in the context of the culture and value systems in which they live and in relation to their goals, expectations, standards and concerns; this definition reflects the view that quality of life refers to a subjective evaluation that is embedded in a cultural, social and environmental context [8]. Health-related quality of life (HQoLife) is generally considered a multidimensional assessment of how disease and treatment affect a patient’s sense of overall function and well-being [9].”*

- <https://doi.org/10.3390/ijtm2040041>

This publication was not included in the reference list. The paragraph was rewritten so that there were no overlapping sentences (above).

3. Please amend your manuscript to include your abstract after the title page.

AUTHORES REPLY: It was corrected. Thank you!

4. Your ethics statement should only appear in the Methods section of your manuscript. If your ethics statement is written in any section besides the Methods, please move it to the Methods section and delete it from any other section. Please ensure that your ethics statement is included in your manuscript, as the ethics statement entered into the online submission form will not be published alongside your manuscript.

AUTHORES REPLY: It was corrected. The topic "Ethics statement" (composed of 2 paragraphs) was properly placed in the end of METHODS section. Thank you!

5. Please include captions for your Supporting Information files at the end of your manuscript, and update any in-text citations to match accordingly. Please see our Supporting Information guidelines for more information: [http://journals.plos.org/plosone/s/supporting-information](http://track.editorialmanager.com/CL0/http:%2F%2Fjournals.plos.org%2Fplosone%2Fs%2Fsupporting-information/1/010f01952a9cd336-271871ee-fc2b-48e0-9bde-f6602fdbc31e-000000/eJbbGqZ5IHUmX-PdXMWUG0AJrhMXPbOcsNe6tE21n1Y=199).

AUTHORES REPLY: Two “In-Text Citations” were included in the manuscript (please see below) and the respective supporting information caption was included after de CONCLUSION Section.

(1) The sentence “The SF36 and EHP30 individual raw scores are found in S1 File” was included in the end of 2^nd^ paragraph of Results section.

(2) The sentence “Details of the linear regression analyses are found in S2 File” was included in the end of 3^rd^ paragraph of Results section.

Supporting information

**S1 File. Quality of life scores (raw data).**

(XLSX)

**S2 File. Details of the linear regression analyses.**

(PDF)

Please review your reference list to ensure that it is complete and correct. If you have cited papers that have been retracted, please include the rationale for doing so in the manuscript text, or remove these references and replace them with relevant current references. Any changes to the reference list should be mentioned in the rebuttal letter that accompanies your revised manuscript. If you need to cite a retracted article, indicate the article’s retracted status in the References list and also include a citation and full reference for the retraction notice.

AUTHORES REPLY: The publications / citations were reviewed; they are correctly positioned in the manuscript. Following reviewers' suggestions, a total of 7 (seven) references were included.

Review Comments to the Author

Reviewer #1:

This manuscript presents the association between endometriosis-related pain duration and burden and poorer psychological well-being and health-related quality of life. Particular attention is paid to comparing the relative burden of different nonmenstrual pelvic symptoms (NMPSymptoms) on health-related quality of life (HRQOL).

AUTHORES REPLY: Thank you very much for your attention.

Reviewer #2:

Thank you for the opportunity to review your submission. Understanding the differential impacts of endometriosis-related symptomatology is an important research and clinical area. I have provided some specific feedback for your consideration below:

General:
1. The tile is quite long. It could be revised for reading ease.

AUTHORES REPLY: Thank you very much for your suggestion. The title has been shortened:

“Non-menstrual pelvic symptoms and women's quality of life: a cross-sectional observational study”.

2. The use of so many acronyms impacts on readability. I do not think you need to abbreviate so many of the terms.

AUTHORES REPLY: Thank you very much for your suggestion. All abbreviations were reviewed. Some were removed; others were modified to make them easier to read. To ensure maximum robustness in the results (which support the study), the abbreviations for the questionnaire scores were maintained in the Methods and Results sections (in parallel with the construction of the tables). However, the use of these details was avoided in the Discussion section.

Introduction:
1. Lines 9-12 - Please include citation for the statement.

AUTHORES REPLY: The Introduction section was rewritten (please see item 2, below); references were included.

2. Your introduction would benefit from a greater emphasis on your study rationale, aims, and hypotheses. These are lacking from the current submission. Your rationale needs more focus on the reasons and potential research/clinical gains for conducting this study.

AUTHORES REPLY: Thank you very much for your suggestion. This study was designed to bring together professionals / scholars from different areas. Thus, keeping the focus on this objective, the Introduction section was rewritten in 6 small paragraphs and 5 references were added in order to improve study rationale, hypotheses and aims.

In short:

Paragraph 1 introduces the condition "endometriosis".

Paragraph 2 introduces the 2 main concepts - pain and quality of life.

Paragraph 3 presents the idea that cyclical pain can evolve into chronic / acyclic pain.

Paragraph 4 highlights the existence of covariates, which can influence the outcome of a treatment (quality of life can be affected not only by painful processes, but also by emotional conditions).

Paragraph 5 highlights the importance of both healthcare professionals and patients understanding / knowing the potential risks and benefits of treatment.

Paragraph 6 highlights the importance of understanding about how different endometriosis pains are for different women and justifies the aim of the study (compare the relative burdens of different non-menstrual symptoms).

Methods:
1. The SF36 table could be moved to supplementary data.

AUTHORES REPLY: Please see below.

2. The EHP-30 table can also be moved to supplementary data file.

AUTHORES REPLY: Obviously, the SF36 and EHP30 tables can be presented as supplementary data. In other words, this will be done if the reviewers deem it best. However, before making this change, we would like to justify the reason for these tables to be in the body of the manuscript.

When we started studying quality of life in patients with endometriosis, we faced difficulties in understanding the domains of the questionnaires, since the studies usually do not present details of the questionnaire used (SF36, EHP30, etc.). Thus, readers cannot consult this information quickly if they are reading articles printed on paper. As stated in item 2 above (regarding Introduction section), this study was designed to bring together professionals / scholars from different areas. Actually, these 2 tables were created with a single objective: to allow (for example) a surgeon to quickly see how the SF36 and EHP30 variables were obtained (even if without a computer).

Results:
1. You have used cross-sectional data and so you need to use the term correlate not predictor.

AUTHORES REPLY: In the Methods section (first submitted version), we had written that "In this study, multiple linear regression models were used not exactly with predictive purposes, but just to compare the relative contribution of independent variables". However, we agree with you that the term "predictor" was not appropriate; we replaced it with "correlate" exactly as suggested. Thank you very much for your attention!

2. Please report your data testing assumptions (e.g., VIF). Did you have to remove any variables because they violated statistical assumptions?

AUTHORES REPLY: In view of the questions, we were forced to add more paragraphs in Methods, Results and Discussion sections. We hope they will be sufficient.

METHODS Section (topic Statistics):

*“…In this study, the statistical models were not used with predictive purposes, but just to compare the relative contribution of explanatory variables (correlates). No explanatory variable was removed (i.e. no backward selection methods) because the objective of the study was not models optimization, but rather to assess the symptoms altogether in a multivariate context. […]. Variance Inflation Factor (VIF) values ≥ 2.5 was conservatively considered indicative of collinearity [41].”*

Results section:

*“Purposely using DDyspareunia, NM Pelvic Pain, NM Dyschezia and NM Dysuria as the only explanatory variables, specific exploratory multiple linear regression models were tested for the 19 different HQoLife domains; results were exposed in Table 4. Statistically, the coefficient of determination (R2) is a measure that represents the proportion of the variance for the outcome that is explained by the correlates; the higher the R2, the better the model fits the data. Its value can only be between 0 and 1, where 0 indicates that the outcome cannot be explained by any of the explanatory variables and 1 indicates that the outcome can be explained without error by them. Most variables did not present a normal distribution (Shapiro-Wilk test). Although theoretically it would be possible to transform the variables to normality, the sample size was considered sufficient for using natural raw data. Importantly, using transformed data makes the comparison of the regression coefficients (B) less natural for most readers - the main objective of this study. In all models tested, VIF values were between 1.06 and 1.21. [Details of the linear regression analyses are found in S2 File.]”*

DISCUSSION Section / Limitations / Assumptions of regression analysis:

*“In this study, a multiple linear regression models (a statistical model that uses a linear equation) was used to explore the relationships between a quantitative dependent variable (HQoLife domain) and 4 quantitative explanatory variables (4 different NM pain symptoms). Regression analysis have several assumptions about the relationship between variables; they include linearity, homoscedasticity and normality. Besides, collinearity (sometimes termed multicollinearity) is usually defined as when two or more independent variables included in the model are highly correlated so that the values of one can be accurately predicted by that of another, which could be a problem.*

*Normality, linearity, and homoscedasticity of variables enhance the analysis even when the statistics are used purely descriptively, as in this study. However, although variables did not present a normal distribution, the Central Limit Theorem reassures us that, with sufficiently large sample sizes, sampling distributions of means are normally distributed regardless of the distributions of variables [70]. In the same way, collinearity was not a problem because all calculated VIF values were between 1.06 and 1.21 – considered problematic when VIF ≥ 2.5 [41].*

*Concerning the cases-to-correlates ratio, required sample size depends on some issues, including the desired power, alpha and beta error levels, number of correlates, and expected effect sizes; conservatively considering a medium-size relationship between the correlates and the dependent variable, alpha = 0.05 and beta = 0.20, the minimum calculated sample size for a model assessing 4 explanatory variables should be 82 individuals [70]. Therefore, the sample size can be considered large enough for what it proposes, that is, to compare the relative burden of 4 different non-menstrual pelvic symptoms on HQoLife.*

*Although it is biologically plausible that any non-menstrual pain causes impairments to HQoLife, no causal relationship should be proposed in this cross-sectional study. As previously highlighted, the statistical models aimed only to compare the covariates.”*

3. The reporting of the regression analyses requires further work. Please discuss the variance explained.

AUTHORES REPLY: The reporting of the regression analyses was now presented in 4 paragraphs - RESULTS Section (see below). Also, details about "the proportion of the variance for the dependent variable that is explained by the explanatory variables" are displayed in the caption to Table 1.

“ […] Purposely using DDyspareunia, NM Pelvic Pain, NM Dyschezia and NM Dysuria as the only explanatory variables, specific exploratory multiple linear regression models were tested for the 19 different HQoLife domains; results were exposed in Table 4. Statistically, the coefficient of determination (R2) is a measure that represents the proportion of the variance for the outcome that is explained by the correlates; the higher the R2, the better the model fits the data. Its value can only be between 0 and 1, where 0 indicates that the outcome cannot be explained by any of the explanatory variables and 1 indicates that the outcome can be explained without error by them. Most variables did not present a normal distribution (Shapiro-Wilk test). Although theoretically it would be possible to transform the variables to normality, the sample size was considered sufficient for using natural raw data. Importantly, using transformed data makes the comparison of the regression coefficients (B) less natural for most readers - the main objective of this study. In all models tested, VIF values were between 1.06 and 1.21. [Details of the linear regression analyses are found in S2 File.]

In this study, although 17 of the 19 tested models were significant and most angular coefficients were positive and statistically different from zero, all models showed a low goodness-of-fit, which is in accord to R2 values taking into account these explanatory variables. The intercept (the constant of the equation) represents the mean value of the outcome when all correlates are equal to zero. In this study, the intercept was a positive value statistically different from zero in all models (P<0.001).

From the perspective of the SF36 questionnaire, NM Pelvic Pain and NM Dyschezia were significant correlates for all HQoLife domains whereas DDyspareunia was a significant correlate for 5 of the 8 SF36 domains (most from the Physical Component Summary). The values of the angular coefficients for DDyspareunia were comparatively smaller than those verified for the NM Pelvic Pain and NM Dyschezia. NM Dysuria was not a significant correlate for any of the SF36 domains.

From the perspective of the EHP30 questionnaire, NM Pelvic Pain and NM Dyschezia were significant correlates for all five domains of the core instrument whereas DDyspareunia was a significant correlate only for 2 of them. The angular coefficients for DDyspareunia were smaller than those observed for NM Pelvic Pain and NM Dyschezia in all models of the core instrument. When the models for each scale of EHP30's modular instrument were tested individually, NM Pelvic Pain was a significant correlate for 4 of the 6 domains, and it was the only significant correlate for the EHP30RwC and EHP30FaT scales. DDyspareunia was a significant correlate for only two of the six domains, and not surprisingly it was the most important correlate for the EHP30Sex. NM Dyschezia was a significant correlate only for EHP30Work, which was also significantly affected by DDyspareunia and NM Pelvic Pain. With a negative value, NM Dysuria was the sole significant correlate for the EHP30SI.”

The first 3 paragraphs of the DISCUSSION Section (topic Overview) address the variance explained (see below).

***“****Overview*

*This study analyzed HQoLife and four distinct and independent non-menstrual pelvic symptoms in consecutive patients with endometriosis referred to a specialized service for candidacy for minimally invasive surgery. Multivariate exploratory analyses indicated that NM Pelvic Pain and NM Dyschezia may be the non-menstrual pelvic symptoms that most impact the HQoLife of women with endometriosis, an association that was observed in practically all domains of both the SF36 and the EHP30 questionnaires.*

*Despite the biological plausibility and statistical significance found in virtually all models, the significant correlates are far from being considered the main determinants of poor HQoLife scores. Indeed, the low explanatory power of the models – evidenced by the weak R2 values – suggests the existence of additional major correlates (i.e. factors not contemplated in the models). Moreover, the presence of a significant positive intercept in all models implies that some HQoLife impairment is expected for any hypothetical woman with endometriosis, even if all four correlates' scores are equal to zero.*

*A possible overestimation of the (weak) variance in HQoLife outcomes attributed to the four non-menstrual pelvic symptoms, however, should not be construed as an error in the interpretation of statistics. Rather, it reflects an intuition that non-menstrual pain does matter, albeit not very consistently in any single case. Thus, one should not be dismissive of modest values for the percentage of the variance in a dependent variable that the Independent (explanatory) variables explain collectively, provided there is statistical assurance that these values are significantly above zero and that the cumulative potential may be substantial [44].”*

4. Please provide a rationale for excluding covariates? I think this creates problems for your results and discussion section.

AUTHORES REPLY: Yes, “not included covariates” is one of the limitations. We have used the available original data. This limitation has been introduced in the RESULTS section and then addressed (from the first paragraphs) in the DISCUSSION section. In fact, exploring this issue has become the core of the manuscript.

In the RESULTS Section (2^nd^ paragraph), we have added: “[…] There was no significant correlation between women's age and any of the domains of the SF36 or EHP30 questionnaires. Thus, the variable "age" was not included in the regression models.”

In the RESULTS Section (3^rd^ paragraph), we have added: *“[…] Purposely using DDyspareunia, NM Pelvic Pain, NM Dyschezia and NM Dysuria as the only explanatory variables, specific exploratory multiple linear regression models were tested for the 19 different HQoLife domains; results were exposed in Table 4. […] [Details of the linear regression analyses are found in S2 File.].”*

In the DISCUSSION section, the topics Overview, Issues beyond acyclic pelvic pain, and Limitations explore/hypothesize precisely the existence of other variables. For example, we added in the last paragraph of the topic Issues beyond acyclic pelvic pain:

*“[…] In this study, the low values of the explanatory coefficients (R2) and the presence of a significant negative constant in the multivariate linear regression models reinforce the concept that many covariates exist, so that chronic pelvic pain deserves interdisciplinary diagnostic and therapeutic approaches.’*

Discussion:
1. The discussion is lacking in depth. There are also several causal statements made throughout and these will need to be reviewed as your data is cross-sectional in nature.

AUTHORES REPLY: The entire manuscript has been reviewed and several passages have been included / changed to enrich the discussion (see track changes file). We think it has become clear that no cause-and-effect relationship has been assumed in this study. Thank you very much for your attention to this fundamental issues!

Reviewer #3:

Dear Authors, congratulations for your article.
Honestly, I believe it is very well written and I have just a few suggestions in order to give insight into some aspects that have not been adequately underlined.
Overall, the structure of the paper is correct and there is a great logicality, making it easily readable.
I only suggest to discuss the potential impact of NM symptoms into the management of patients potentially facing the need for surgery when thriving for pregnancy (<http://dx.doi.org/10.3390/jcm13237349>).

AUTHORES REPLY: In view of this question, being very careful not to delve too deeply into the topics “surgery” and "assisted reproductive technology", we added:

“DISCUSSION Section / 2^nd^ paragraph of the topic What is this study for? Interpretation / Generalizability:

*[…] A better understanding of the potential impact of different types of non-menstrual pelvic pain on HQoLife can help clinicians and patients choose one treatment strategy versus another (including assisted reproductive technology versus surgery in endometriosis-related infertility) because the planning must be individualized according to patient symptoms [29].”*

*The reference (<http://dx.doi.org/10.3390/jcm13237349>) was included: [29] Colombi I, Ginetti A, Cannoni A, Cimino G, d'Abate C, Schettini G, Giorgi M, Raimondo D, Martire FG, Lazzeri L, Zupi E, Centini G. Combine Surgery and In Vitro Fertilization (IVF) in Endometriosis-Related Infertility: When and Why. J Clin Med. 2024;13(23):7349. doi.org/10.3390/jcm13237349.

Eventually, which do you think is be the impact of CS in this kind of symptoms (<http://dx.doi.org/10.1016/j.jmig.2022.10.007>)?

AUTHORES REPLY: Objectively, we think this is a fact. Although the comment was very encouraging, we have added just one paragraph (with 5 references) in the DISCUSSION section - topic Issues beyond acyclic pelvic pain (see below):

*“Very recently, nociplastic pain has entered the medical vernacular to describe pain conditions that are neither neuropathic (caused by nerve damage) nor nociceptive (caused by ongoing inflammation and damage of tissues), but commonly experienced by people worldwide [67]. Nociplastic pain may be a consequence of central sensitization - a highly prevalent condition that also includes chronic pain, allodynia, hypersensitivity, hyperalgesia, mood disturbances and depression [68]. Although the mechanisms that underlie this type of pain are not entirely understood [67], identifying patients whose pain is complicated by central nervous system sensitization may be the key problem when conventional treatment does not completely alleviate the pain [69]. Central sensitization may be defined operationally as amplification of neural signaling within the central nervous system that elicits pain hypersensitivity [70]; this condition has often been overlooked in patients with endometriosis, as well as the need for a multidisciplinary, multimodal approach to endometriosis-related pain [71]. The presence of non-menstrual symptoms does suggest some degree of central sensitization, which may negatively impact HQoLife. In this study, the low values of the explanatory coefficients (R2) and the presence of a significant negative constant in the multivariate linear regression models reinforce the concept that many covariates exist, so that chronic pelvic pain deserves interdisciplinary diagnostic and therapeutic approaches.”*

*The reference (<http://dx.doi.org/10.1016/j.jmig.2022.10.007>) was included: [68] Raimondo D, Raffone A, Renzulli F, Sanna G, Raspollini A, Bertoldo L, Maletta M, Lenzi J, Rovero G, Travaglino A, Mollo A, Seracchioli R, Casadio P. Prevalence and Risk Factors of Central Sensitization in Women with Endometriosis. J Minim Invasive Gynecol. 2023;30(1):73-80.e1. doi: 10.1016/j.jmig.2022.10.007.

The 10 references below were included:

[11] Velho RV, Werner F, Mechsner S. Endo Belly: What Is It and Why Does It Happen?-A Narrative Review. J Clin Med. 2023;12(22):7176. doi: 10.3390/jcm12227176.

[12] Romão AP, Gorayeb R, Romão GS, Poli-Neto OB, dos Reis FJ, Rosa-e-Silva JC, Barbosa Hde F, Nogueira AA. Chronic pelvic pain: multifactorial influences. J Eval Clin Pract. 2011;17(6):1137-9. doi: 10.1111/j.1365-2753.2010.01485.x.

[13] Drabble SJ, Long J, Alele B, O'Cathain A. Constellations of pain: a qualitative study of the complexity of women's endometriosis-related pain. Br J Pain. 2021;15(3):345-356. doi: 10.1177/2049463720961413.

[29] Colombi I, Ginetti A, Cannoni A, Cimino G, d'Abate C, Schettini G, Giorgi M, Raimondo D, Martire FG, Lazzeri L, Zupi E, Centini G. Combine Surgery and In Vitro Fertilization (IVF) in Endometriosis-Related Infertility: When and Why. J Clin Med. 2024;13(23):7349. doi: 10.3390/jcm13237349.

[30] Della Corte L, Di Filippo C, Gabrielli O, Reppuccia S, La Rosa VL, Ragusa R, Fichera M, Commodari E, Bifulco G, Giampaolino P. The Burden of Endometriosis on Women's Lifespan: A Narrative Overview on Quality of Life and Psychosocial Wellbeing. Int J Environ Res Public Health. 2020;17(13):4683. doi: 10.3390/ijerph17134683.

[78] Guillemot C, Klinkenberg J, Sordes F. The psychopathological repercussions on patients faced with pain: A focus on endometriosis. Encephale. 2024;50(3):289-295. doi: 10.1016/j.encep.2023.06.019.

[67] Fitzcharles MA, Cohen SP, Clauw DJ, Littlejohn G, Usui C, Häuser W. Nociplastic pain: towards an understanding of prevalent pain conditions. Lancet. 2021;397(10289):2098-2110. doi: 10.1016/S0140-6736(21)00392-5.

[68] Raimondo D, Raffone A, Renzulli F, Sanna G, Raspollini A, Bertoldo L, Maletta M, Lenzi J, Rovero G, Travaglino A, Mollo A, Seracchioli R, Casadio P. Prevalence and Risk Factors of Central Sensitization in Women with Endometriosis. J Minim Invasive Gynecol. 2023;30(1):73-80.e1. doi: 10.1016/j.jmig.2022.10.007.

[69] Orr NL, Wahl KJ, Lisonek M, Joannou A, Noga H, Albert A, Bedaiwy MA, Williams C, Allaire C, Yong PJ. Central sensitization inventory in endometriosis. Pain. 2022 Feb 1;163(2):e234-e245. doi: 10.1097/j.pain.0000000000002351.

[70] Woolf CJ. Central sensitization: implications for the diagnosis and treatment of pain. Pain. 2011;152(3 Suppl):S2-S15. doi: 10.1016/j.pain.2010.09.030.

[71] Cetera GE, Merli CEM, Vercellini P. A Multimodal Approach to Symptomatic Endometriosis: A Proposed Algorithm for Clinical Management. Reprod Sci. 2025;32(2):289-299. doi: 10.1007/s43032-024-01763-w.
